# Exploring the factor structure of the Food Cravings Questionnaire-Trait in Cuban adults

**DOI:** 10.3389/fpsyg.2014.00214

**Published:** 2014-03-18

**Authors:** Boris C. Rodríguez-Martín, Osana Molerio-Pérez

**Affiliations:** Department of Psychology, Faculty of Psychology, Central University “Marta Abreu” of Las VillasSanta Clara, Cuba

**Keywords:** assessment, food cravings, factor analysis, reliability, food cravings questionnaire-trait, elaborated intrusion theory of desire

## Abstract

Food cravings refer to an intense desire to eat specific foods. The Food Cravings Questionnaire-Trait (FCQ-T) is the most commonly used instrument to assess food cravings as a multidimensional construct. Its 39 items have an underlying nine-factor structure for both the original English and Spanish version; but subsequent studies yielded fewer factors. As a result, a 15-item version of the FCQ-T with one-factor structure has been proposed (FCQ-T-reduced; see this Research Topic). The current study aimed to explore the factor structure of the Spanish version for both the FCQ-T and FCQ-T-reduced in a sample of 1241 Cuban adults. Results showed a four-factor structure for the FCQ-T, which explained 55% of the variance. Factors were highly correlated. Using the items of the FCQ-T-reduced only showed a one-factor structure, which explained 52% of the variance. Both versions of the FCQ-T were positively correlated with body mass index (BMI), scores on the Food Thoughts Suppression Inventory and weight cycling. In addition, women had higher scores than men and restrained eaters had higher scores than unrestrained eaters. To summarize, results showed that (1) the FCQ-T factor structure was significantly reduced in Cuban adults and (2) the FCQ-T-reduced may represent a good alternative to efficiently assess food craving on a trait level.

## Introduction

Food craving is a motivational state, defined as an intense desire to eat specific foods (Tiggemann and Kemps, 2005). It is a common experience in everyday life for the majority of individuals. However, frequent experiences of food craving are associated with over- or binge eating (Kemps and Tiggemann, 2010; Havermans, 2013). Additionally, experiencing food cravings habitually could be a psychological factor that contributes to diet failure (Meule et al., 2012).

Cross-cultural studies have shown differences in the type of food cravings (Hormes and Rozin, 2010). Because of this, those instruments which assess general responses to food cues could be more useful than those which target specific foods. In order to assess habitual food cravings, the Food Cravings Questionnaire-Trait (FCQ-T) was designed (Cepeda-Benito et al., 2000b). This is the most extensively validated and adapted food craving measure, currently available in English, Spanish, Dutch, Korean and German (Cepeda-Benito et al., 2000a,b, 2003; Nijs et al., 2007; Rodríguez et al., 2007b; Noh et al., 2008; Meule et al., 2012).

The FCQ-T also measures craving for specific foods, but those are not explicitly pre-defined as in other questionnaires such as the Food Craving Inventory (White et al., 2002). The FCQ-T assesses food cravings as a multidimensional construct, divided in nine subscales (Cepeda-Benito et al., 2000a): intentions and plans to consume food; anticipation of positive reinforcement that may result from eating; anticipation of relief from negative states and feelings as a result of eating; possible lack of control over eating if food is eaten; thoughts or preoccupation with food; craving as a physiological state; emotions that may be experienced before or during food cravings or eating; environmental cues that may trigger food cravings; and guilt that may be experienced as a result of cravings and/or giving into them.

Scores on the FCQ-T have been positively related to body mass index (BMI) (Meule et al., 2012), sensitivity to reward (Franken and Murris, 2005), rigid dietary control strategies (Meule et al., 2011), obesity (Vander-Wal et al., 2007), eating disorder symptoms (Cepeda-Benito et al., 2003; Moreno et al., 2008) and food addiction symptoms (Meule and Kübler, 2012).

Another important variable that has not been investigated with the FCQ-T yet is food thoughts suppression (Barnes and Tantleff-Dunn, 2010). According to the Elaborated Intrusion Theory of Desire (EI-Theory), elaboration of unwanted intrusive thoughts about a desired target is a gateway which leads to cravings (Kavanagh et al., 2005). An intrusive thought could emerge from an associative process linked with: physiological deficit, negative affect, external cues, other cognitive activity and anticipatory responses to the target; but their progressive elaboration is the key process for the cravings' maintenance.

Food-related thoughts may play an important role in the maintenance of unhealthy eating behaviors and the suppression of these thoughts could provoke increased consumption of the desired food (May et al., 2012). Furthermore, there is evidence that supports the futility of the intention to suppress food-related thoughts to control food cravings in real life settings (Rodríguez-Martín et al., 2013). However, it is important to highlight that some individuals are more vulnerable to both intrusive thoughts about food and food cravings, even if they are not attempting to suppress them (May et al., 2012).

Although both the English and Spanish version of the FCQ-T yielded nine dimensions of food cravings, analyses of the factor structure of the FCQ-T showed fewer factors for the German and Dutch version (Nijs et al., 2007; Meule et al., 2012), as well as in overweight and obese individuals (Vander-Wal et al., 2007), including bariatric surgery candidates (Crowley et al., 2014). As a result, two reduced versions have been proposed: a 21-item version for assessing general food craving on a trait level (Nijs et al., 2007) and a 15-item version of the FCQ-T (FCQ-T-reduced) with a one-factor structure (Meule et al., 2014).

One possible explanation for these variations could be the difficulties encountered when translating the term craving into other languages. The majority of native speakers of 20 languages generally agreed that whatever translation they provided, was not completely adequate for capturing the meaning of the word *craving* (Hormes and Rozin, 2010). In fact, meanings could change among native speakers from the same language in different countries. For example craving could be translated in Spanish as *ansia* (Rodríguez et al., 2007a) or *antojo* (Cepeda-Benito et al., 2000a), but in Cuba many individuals mainly use some expressions such as “anxiety to eat” [ansiedad de comer], to refer to a strong desire to eat. In this context, *anxiety* does not refer to an emotion that leads to eating (Macht, 2008)^1^, instead it means craving^2^. Some researchers are currently using the term *craving* in Spanish without translation (González and Donaire, 2012; Jáuregui-Lobera et al., 2012a,b).

Another explanation could be the context itself. Cubans prefer sweet and fatty foods over fruits and vegetables (Porrata-Maury, 2009), which is consistent with the food preferences generally associated with Western culture (Cepeda-Benito et al., 2000a); but our food environment could be quite different to the Spanish. Extreme difficulties experienced in Cuba between 1990 and 1995, known as the *special period*, conditioned a decrease of food availability for the majority of the population. For example, Cubans consumed approximately 1863 kcal of food per day during 1993 (Jiménez-Acosta et al., 1998), which is a very low amount of energy intake taking into account that participants of the well-known Minnesota Starvation Experiment consumed 1800 kcal of food per day during 6 months in 1945 (Kalm and Semba, 2005). After the *special period*, data from a national survey conducted during 2001 showed that overweight and obesity rates increased rapidly (Jiménez-Acosta et al., 2012). As it has been suggested the perception of harshness could promote overeating (Laran and Salerno, 2013) and individuals would be less motivated to exert their self-control (Hoffman and Kotabe, 2012).

Language and environment could influence the way food craving is experienced in everyday life. However, it is important to highlight that the nine-factor structure was obtained by confirmatory factor analysis (CFA) (Cepeda-Benito et al., 2000a,b; Moreno et al., 2008) while divergent factor structures were obtained by exploratory factor analysis (Nijs et al., 2007; Vander-Wal et al., 2007; Meule et al., 2012; Crowley et al., 2014; Meule et al., 2014). Exploratory factor analysis (EFA) is technically different from CFA: the first is used for theory-building, whereas the second is used primarily for theory-testing (Matsunaga, 2010). Because of this, it is necessary to explore the factor structure, validity and reliability of the Spanish version of FCQ-T and FCQ-T-reduced among Cuban adults.

As a first step a CFA was performed to test whether the data fit into the nine subscales of the Spanish FCQ-T as found by Cepeda-Benito et al. (2000a). A second step was to perform principal component analyses (PCA) on both the FCQ-T and FCQ-T-reduced, to analyze the resulting components, taking into account the particularities of the Cuban context previously described. PCA is considered as an effective tool to reduce a pool of items into a smaller number of components with loss of as little information as possible, (Matsunaga, 2010), whereas the number of factors was determined with parallel analysis (Hayton et al., 2004).

According to previous results, it was expected that PCA show a number of factors less than nine for the FCQ-T. With regard to reliability and validity indices, we expected for both versions high internal consistency, positive correlations with BMI, weight cycling and higher scores in restrained eaters and women as compared to unrestrained eaters and men. Finally, according to the EI-theory's prediction, a strong correlation between food cravings and food thoughts suppression was also expected.

## Materials and methods

### Participants

Sample characteristics are displayed in Table 1. Participants were 1241 individuals from the general population, who were between 18 and 64 years old (*M* = 32.57, *SD* = 12.88), with 68.7% being females. Regarding marital status, most of the participants 53.1% were single while 41.6% were married. In addition, 63.4% had obtained secondary education. The majority of participants were classified as *healthy* by the Cuban National Health Care System^3^ (see Procedures section). Finally, BMI ranged between 18.52 and 39.47 kg/m^2^ (*M* = 26.06, *SD* = 4.14). BMI was additionally classified according to standard guidelines (WHO, 2011) as normal weight (BMI = 18.50–24.99 kg/m^2^), overweight (BMI = 25.00–29.99 kg/m^2^) and obese (BMI > 30.00 kg/m^2^).

**Table 1:** **Socio-demographic and clinical data of the sample**.

**Variable**	**Class**	**Freq**.	**%**
Gender	Female	852	68.7
	Male	389	31.3
BMI	Normal weight	520	41.9
	Overweight	520	41.9
	Obese	201	16.2
Education	Primary	74	6.0
	Secondary	785	63.4
	Higher	380	30.7
Marital Status	Single	659	53.1
	Married	516	41.6
	Divorced	58	4.7
	Widow	8	0.6
Health condition	Healthy	958	77.2
	Asthma	77	6.2
	High blood pressure	80	6.4
	Diabetes	12	1.0
	Others	114	9.2

Exclusion criteria included pregnancy, lactation, active eating disorders (Vander-Wal et al., 2007) or any diagnosed psychopathological disorder. Older adults (≥65 years) were also excluded, as it has been observed that there are changes at this stage not only in the amount or type of food and nutrients they consume, but in the way they think about food (Elsner, 2003).

### Measures

#### Socio-demographic/anthropometric and clinical data

Participants were asked to provide age; gender; height; education level; marital status and current weight. Finally, clinical and psychopathological diagnoses were retrieved from medical records.

#### Restrained eating was assessed using a single item

Do you often restrain your food intake to reduce or maintain your weight? (Yes/No)

#### Weight cycling was assessed using 3 items from the weight cycling questionnaire, with a cronbach's α = 0.76 (Rodríguez-Martín et al., 2012b)

(1) “How often are you a yo-yo dieter?”; (2) “How often do you start a diet and quit?” and (3) “How often do you regain more weight than you lost on a diet?” The Weight Cycling Questionnaire is a brief assessment of individuals' tendency to experience weight fluctuations (Peterson, 2008). Individuals respond to questions on a 5-point Likert scale ranging from 1 (never) to 5 (always). Higher scores represent a history of more diet failures.

#### Food cravings questionnaire-trait

The Spanish version of the questionnaire (Cepeda-Benito et al., 2000a) measures the intensity of nine trait dimensions of food cravings (see Introduction section). Instructions asked participants how frequently each statement “would be true for you in general” using a 6-point scale that ranged from 1 (*never* or *not applicable*) to 6 (*always*).

#### Food cravings questionnaire-trait-reduced

This is a 15-item version of the FCQ-T (Meule et al., 2014). Selected items were those with the highest item-total-correlations in the German FCQ-T validation study (Meule et al., 2012) and belonged to subscales from the Spanish version (Cepeda-Benito et al., 2000a; Moreno et al., 2008) *lack of control over eating* (items 2,3,25,26,29), *thoughts or preoccupation with food* (items 6,8,27, 31, 32), *intentions and plans to consume food* (items 5,18), *emotions before or during food craving* (items 20,33), and *cues that may trigger food craving* (item 35).

#### Food thought suppression inventory

This 15-item inventory was created based on the White Bear Suppression Inventory (Wegner and Zanakos, 1994), as a measure of food thought suppression (Barnes et al., 2009; Barnes and White, 2010). It was validated for a Cuban sample with a Cronbach's α = 0.95 (Rodríguez-Martín et al., 2012a). Participants respond to questions such as, “There are foods that I try not to think about” on a 5-point Likert scale ranging from 1 (*strongly disagree*) to 5 (*strongly agree*).

### Procedures

The study was approved by the Scientific Council at the authors' institution. Forty Psychology students called *surveyors* were trained for the sample selection and assessment (16 h at the authors' institution). Training included lectures about overweight and obesity, eating behavior and food cravings, as well as practical sessions on data collection. All the characteristics of the study were explained to them and they were also instructed to contact the main researcher in case of further doubts. After that, each surveyor was assigned to a supervisor from a health care institution from their municipality.

The sample selection was carried out in each surveyor's *health area* by inviting members of the corresponding community to participate through verbal announcements. All participants were visited in-person, informed about the study procedures by a surveyor, and signed an informed consent prior to assessment. Surveyors were asked to assess individuals who agreed to previously complete the corresponding measurements of weight and height at their physician's office and fulfilled the inclusion criteria after the revision of their medical records. The time required to complete the questionnaires never exceeded 45 min. All the participants voluntarily accepted to participate in the study and no compensation was offered to them.

### Statistical analyses

CFA was performed with AMOS version 18 using the maximum-likelihood estimation method. The model fit was evaluated with the same fit indices reported by Cepeda-Benito et al. (2000b), which were: the χ^2^ statistic; the Goodness-of-Fit Index (GFI); the Normed-Fit Index (NFI); the Tucker-Lewis Index (TLI); the Comparative Fit Index (CFI), and the Root Mean Square Error of Approximation (RMSEA). For the GFI, NFI, TLI, and CFI, values of approximately 0.90 or greater reflect an adequate fit (Byrne, 1989; Mulaik et al., 1989). Finally, values of the RMSEA of 0.05 or less indicate a close fit, values between 0.05 and 0.08 indicate adequate fit, and values greater than 0.10 indicate need for improvement in the model (Browne and Cudeck, 1993). All other analyses were performed with SPSS version 20. PCA was performed on both the FCQ-T and the FCQ-T-reduced, using an oblique rotation (Promax, κ = 4) because correlations between factors were expected. The number of factors was determined with parallel analysis (Hayton et al., 2004), which is considered one of the most accurate factor retention methods (Matsunaga, 2010). The *Kaiser-Meyer-Olkin Measure of Sampling Adequacy* and *Bartlett's Test of Sphericity* were used in order to test if data met requirements for exploratory factor analysis.

As a measure for internal consistency, Cronbach's α was determined for the full version of each questionnaire, as well as for the factors of the FCQ-T. Additionally, to examine the construct validity of each version, Pearson correlations were calculated between FCQ-T and FCQ-T-reduced scores and age, BMI, and scores on the Weight Cycling Questionnaire and Food Thought Suppression Inventory. Values of *r* above 0.1,0.3, and 0.5 were interpreted as small, medium and large effect sizes, respectively (Sink and Mvududu, 2010). Finally, differences in scores of both the FCQ-T and FCQ-T-reduced between men and women and between restrained and unrestrained eaters were tested with *t*-tests. Effect sizes were calculated with Cohen's *d* from *t*-test values using ViSta 7, where values of 0.2,0.5, and 0.8 were defined as small, medium and large effects, respectively (Ledesma et al., 2009). All statistical tests are reported two-tailed and *p*-values marked as *ns* refer to *p* = 0.05.

## Results

### FCQ-T

The CFA fit indices of the FCQ-T were as follows: χ^2^_(90)_ = 1159.729, *p* < 0.001; GFI = 0.79; NFI = 0.84; TLI = 0.84; CFI = 0.86; and RMSEA = 0.07. Except for the RMSEA, all indices did not suggest a good fit for the nine-factor structure of the FCQ-T.

**Figure 1:**
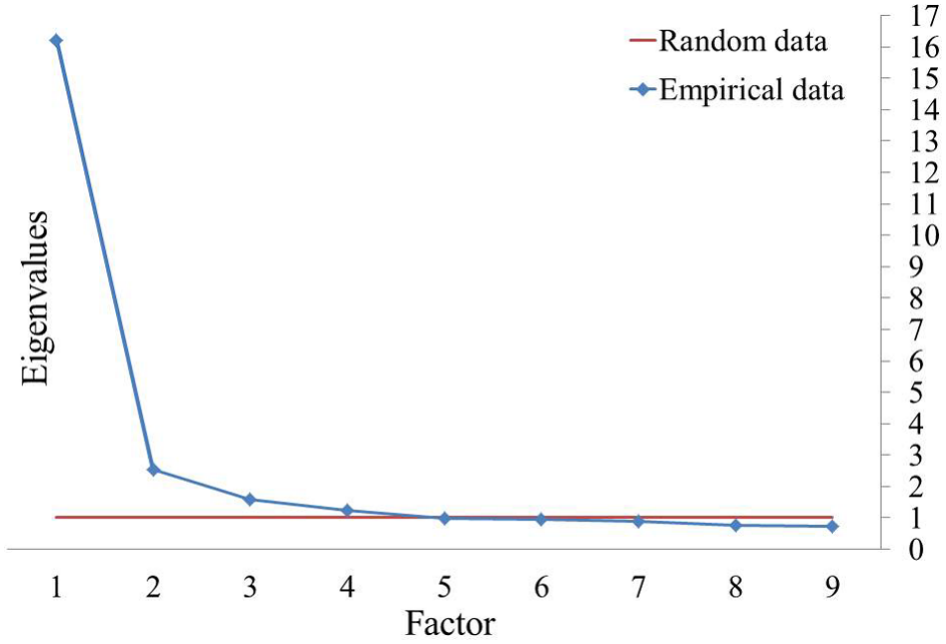
**Scree plot and parallel analysis of eigenvalues in FCQ-T**.

The Kaiser-Meyer-Olkin Measure of Sampling Adequacy (KMO = 0.97) and Bartlett's Test of Sphericity [χ^2^_(741)_ = 27727.31, *p* < 0.001] indicated that the data were adequate for conducting a PCA. Scree plot and parallel analysis indicated a four-factor structure (Figure 1), which explained 55.30% of variance. Eigenvalues before rotation were 16.2, 2.5, 1.6, and 1.2 and after rotation were 13.33, 10.91, 9.89, and 10.39.

A visual inspection of Table 2 shows that our four-factor solution was not merely a combination of the nine original subscales. Factor 1 grouped items of *thoughts about food*, *intentions to consume food*, *guilt from cravings, lack of control* and *positive reinforcement*. Factor 2 included items of *lack of control over eating*, *cues that trigger food cravings* and one item of *guilt from cravings*. Factor 3 included items of *craving as hunger*, *anticipation of positive reinforcement from eating* and one item of *intentions to consume food*. Factor 4 included items of *emotions experienced during food cravings* and *anticipation of relief from negative states and feelings as a result of eating*.

**Table 2:** **Factor loadings and item statistics of the food craving questionnaire trait**.

**Item**	**Factor**				**Description**	**Initial factor**	**Mean (*SD*)**	**r_item-total correlation_**
	**1**	**2**	**3**	**4**				
5	**0.528**	0.238	0.140	−0.067	Sin duda alguna, las ganas de comer me hacen pensar cómo voy a conseguirlo. [Food cravings invariably make me think of ways to get what I want to eat.]	Intentions to eat	2.98 (1.87)	0.708^**^
6	**0.778**	0.140	−0.143	0.000	No hago más que pensar en la comida. [I feel like I have food on my mind all the time]	Food-related thoughts	2.29 (1.56)	0.689^**^
7	**0.618**	0.246	−0.104	0.002	A menudo me siento culpable cuando deseo ciertas comidas. [I often feel guilty for craving certain foods]	Guilty feelings	2.61 (1.82)	0.666^**^
8	**0.778**	−0.151	0.089	0.008	A veces me encuentro pensativo preocupado con comida. [I find myself preoccupied with food]	Food-related thoughts	2.58 (1.73)	0.638^**^
9	**0.530**	−0.264	0.300	0.167	Como para sentirme mejor. [I eat to feel better]	Positive reinforcement	2.96 (1.81)	0.612^**^
10	**0.502**	−0.234	0.479	−0.018	Algunas veces, mi vida parece perfecta cuando como lo que me apetece. [Sometimes, eating makes things seem just perfect]	Positive reinforcement	3.41 (1.91)	0.605^**^
17	**0.499**	0.312	−0.159	0.042	Cuando como algo que deseo con intensidad me siento culpable. [When I eat what I am craving I feel guilty about myself]	Guilty feelings	2.61 (1.89)	0.606^**^
18	**0.415**	0.081	0.314	0.013	Cada vez que deseo comer algo en particular me pongo a hacer planes para comer. [Whenever I have cravings, I find myself making plans to eat]	Intentions to eat	3.22 (1.89)	0.676^**^
27	**0.805**	0.232	−0.148	−0.060	Por mucho que lo intento, no puedo parar de pensar en comer. [I can't stop thinking about eating no matter how hard I try]	Food-related thoughts	2.30 (1.66)	0.732^**^
28	**0.818**	0.102	−0.151	−0.024	Gasto demasiado tiempo pensando en lo próximo que voy a comer. [I spend a lot of time thinking about whatever it is I will eat next]	Food-related thoughts	2.16 (1.60)	0.667^**^
29	**0.582**	0.377	−0.079	−0.012	Si me dejo llevar por la tentación de comer pierdo todo mi control. [If I give in to a food craving, all control is lost]	Lack of control	2.53 (1.85)	0.743^**^
30	**0.901**	−0.024	−0.099	−0.110	A veces me doy cuenta de que estoy soñando despierto y estoy soñando en comer. [I daydream about food]	Food-related thoughts	2.16 (1.60)	0.612^**^
31	**0.528**	0.322	0.185	−0.133	Cada vez que se me antoja una comida sigo pensando en ella hasta que me la como. [Whenever I have a food craving, I keep on thinking about eating until I actually eat the food]	Food-related thoughts	3.08 (1.88)	0.755^**^
32	**0.485**	0.288	0.174	−0.044	Cuando tengo muchas ganas de comer algo estoy obsesionado con comerlo. [If I am craving something, thoughts of eating it consume me]	Food-related thoughts	3.02 (1.86)	0.752^**^
1	0.189	**0.356**	0.152	0.071	Cuando estoy con alguien que esta comiendo me entra hambre. [Being with someone who is eating often makes me hungry]	Cue-depending Eating	3.47 (1.80)	0.619^**^
2	0.259	**0.523**	−0.024	0.077	Cuando tengo deseos intensos de comer, una vez que empiezo no puedo parar. [When I crave something, I know I won't be able to stop eating once I start]	Lack of control	3.01 (1.84)	0.687^**^
3	0.056	**0.551**	0.203	0.048	A veces, cuando como lo que se me antoja, pierdo control y como demasiado. [If I eat what I am craving, I often lose control and eat too much]	Lack of control	3.86 (1.85)	0.680^**^
4	0.054	**0.669**	−0.014	0.124	Detesto no poder resistir la tentación de comer. [I hate it when I give into cravings]	Guilty feelings	3.36 (1.99)	0.671^**^
22	−0.218	**0.558**	0.491	0.020	Si tengo la comida que deseo, no puedo resistir la tentación de comerla. [If I get what I am craving I cannot stop myself from eating it]	Lack of control	4.37 (1.74)	0.639^**^
25	0.120	**0.591**	0.153	0.015	No tengo la fuerza de voluntad de resistir mis deseos de comer lo que se me antoja. [I have no will power to resist my food crave]	Lack of control	3.54 (1.99)	0.704^**^
26	0.462	**0.485**	−0.102	0.028	Una vez que me pongo a comer tengo problemas en dejar de comer. [Once I start eating, I have trouble stopping]	Lack of control	2.79 (1.87)	0.737^**^
34	−0.069	**0.602**	0.206	0.048	Cada vez que voy a un banquete termino comiendo más de lo que necesito. [Whenever I go to a buffet I end up eating more than what I needed]	Cue-depending Eating	3.93 (1.94)	0.527^**^
35	−0.044	**0.665**	0.238	0.013	Es difícil resistir la tentación de tomar comidas apetecibles que están a mi alcance. [It is hard for me to resist the temptation to eat appetizing foods that are within reach]	Cue-depending eating	3.91 (1.94)	0.680^**^
36	0.230	**0.456**	−0.017	0.211	Cuando estoy con alguien que se pasa comiendo, yo también me paso. [When I am with someone who is overeating, I usually overeat too]	Cue-depending eating	3.10 (1.93)	0.717^**^
11	−0.209	0.186	**0.706**	−0.036	Se me hace la boca agua cuando pienso en mis comidas favoritas. [Thinking about my favorite foods makes my mouth water]	Feelings of hunger	4.69 (1.69)	0.474^**^
12	−0.202	0.220	**0.709**	−0.114	Siento deseos intensos de comer cuando mi estómago está vacío. [I crave foods when my stomach is empty]	Feelings of hunger	4.87 (1.57)	0.446^**^
13	0.164	0.102	**0.592**	−0.086	Siento como que mi cuerpo me pidiera ciertas comidas. [I feel as if my body asks me for certain food]	Feelings of hunger	4.00 (1.73)	0.607^**^
14	0.262	0.048	**0.404**	0.005	Me entra tanta hambre que mi estómago se siente como un pozo sin fondo. [I get so hungry that my stomach seems like a bottomless pit]	Feelings of hunger	3.49 (1.79)	0.580^**^
15	−0.019	−0.013	**0.783**	−0.013	Cuando como lo que deseo me siento mejor. [Eating what I crave makes me feel better]	Positive reinforcement	4.35 (1.71)	0.451^**^
16	0.321	−0.228	**0.359**	0.314	Cuando como lo que deseo me siento menos deprimido. [When I satisfy a craving I feel less depressed]	Feelings of relief	3.06 (1.85)	0.615^**^
23	−0.058	0.317	**0.616**	−0.047	Cuando se me antoja una comida, intento comerla tan pronto como pueda. [When I crave certain foods, I usually try to eat them as soon as I can]	Intentions to eat	4.10 (1.75)	0.628^**^
24	0.008	0.092	**0.708**	−0.025	Comer lo que me apetece mucho me sienta estupendamente. [When I eat what I crave I feel great]	Positive reinforcement	4.14 (1.76)	0.593^**^
19	0.321	−0.124	0.237	**0.401**	El comer me tranquiliza. [Eating calms me down]	Feelings of relief	3.09 (1.78)	0.675^**^
20	−0.093	0.238	−0.007	**0.662**	Siento deseos de comer cuando estoy aburrida, enfadada, o triste. [I crave foods when I feel bored, angry, or sad]	Negative affect	3.19 (1.85)	0.622^**^
21	−0.250	0.146	0.122	**0.680**	Después de comer no tengo tanta ansiedad. [I feel less anxious after I eat]	Feelings of relief	3.40 (1.83)	0.522^**^
33	0.182	0.132	−0.129	**0.585**	A menudo deseo comer cuando siento emociones fuertes. [My emotions often make me want to eat]	Negative affect	2.72 (1.79)	0.613^**^
37	0.297	−0.080	0.214	**0.442**	Comer me alivia. [When I eat food, I feel comforted]	Positive reinforcement	3.10 (1.81)	0.699^**^
38	−0.095	0.210	−0.128	**0.798**	Cuando estoy muy estresada me entran deseos fuertes de comer. [When I'm stressed out, I crave food]	Negative affect	3.07 (1.85)	0.610^**^
39	0.089	−0.013	−0.221	**0.743**	Me entran deseos fuertes de comer cuando estoy disgustado. [I crave foods when I'm upset]	Negative affect	2.24 (1.61)	0.484^**^

** *p < 0.001*.

Item difficulties ranged between 2.16 and 4.87, with highest mean scores grouped within Factor 3 (from 3.06 to 4.87, see Table 2). Range of item-total-correlations was *r* = 0.451–0.755 (Table 2) and factors were highly correlated with each other as with the FCQ-T total score (Table 3). Internal consistency was Cronbach's α = 0.92 for the FCQ-T total score and for the subscales were as follow: α_F1_ = 0.93, α_F2_ = 0.91, α_F3_ = 0.85, and α_F4_ = 0.84.

**Table 3:** **Correlations between study variables**.

	**Mean (*SD*)**	**FCQ-T-r**	**FCQ-T**	**F1_FCQ-T_**	**F2_FCQ-T_**	**F3_FCQ-T_**	**F4_FCQ-T_**	**Age**	**BMI**	**WCQ**
FCQ-T-r	45.02 (19.74)									
FCQ-T	120.81 (44.70)	973^**^								
F1_FCQ-T_	37.92 (18.12)	0.942^**^	0.937^**^							
F2_FCQ-T_	35.34 (14.01)	0.907^**^	0.917^**^	0.782^**^						
F3_FCQ-T_	32.70 (9.74)	0.696^**^	0.789^**^	0.651^**^	0.702^**^					
F4_FCQ-T_	20.79 (8.96)	0.807^**^	0.854^**^	0.756^**^	0.707^**^	0.605^**^				
Age	32.57 (12.88)	0.191^**^	0.182^**^	0.186^**^	0.177^**^	0.145^**^	0.117^**^			
BMI	26.06 (4.14)	0.346^**^	0.324^**^	0.292^**^	0.361^**^	0.196^**^	0.252^**^	0.521^**^		
WCQ	4.98 (2.87)	0.232^**^	0.219^**^	0.190^**^	0.251^**^	0.079^**^	0.210^**^	0.040 ns	0.276^**^	
FTSI	39.68 (17.82)	0.733^**^	0.720^**^	0.736^**^	0.660^**^	0.499^**^	0.555^**^	0.197^**^	0.330^**^	0.240^**^

FCQ-T, Food Craving Questionnaire-Trait; FCQ-T-r, Food Craving Questionnaire-Trait-reduced; F1_FCQ-T_, Factor 1 of the Food Craving Questionnaire-Trait [Σitems 5–10, item 17, item 18, item 27–32]; F2_FCQ-T_, Factor 2 of the Food Craving Questionnaire-Trait [Σitem 1–4, item 22, item 25, item 26, item 34-36]; F3_FCQ-T_, Factor 3 of the Food Craving Questionnaire-Trait [Σitems 11–16, item 23, item 24]; F4_FCQ-T_, Factor 4 of the Food Craving Questionnaire-Trait [Σitems 19-21, item 33, item 37–39]; BMI, Body Mass Index; WCQ, Weight Cycling Questionnaire; FTSI, Food Thoughts Suppression Inventory;

** *p < 0.001*.

Regarding construct validity of the FCQ-T, results are displayed in Table 2. FCQ-T total scores showed positive correlations with age (small effect size), weight cycling (from small to medium effect size), BMI (medium effect size) and food thoughts suppression (large effect size). Scores of Factor 1 and Factor 2 showed the same pattern as FCQ-T total scores, but in Factor 3 and Factor 4 the effect sizes tended to decrease and were considered as small for weight cycling and from small to medium for BMI. The effect size of food thoughts suppression was large either for the FCQ-T total score or for each subscale.

Restrained eaters had higher scores on food craving and all its dimensions than unrestrained eaters (Table 4). Women had significantly higher scores than men, except scores on Factor 3 (Table 4). Effect sizes could be generally considered small for gender and from small to medium for restrained eating.

**Table 4:** **Food cravings comparisons acording to gender and restrained eating**.

	**Gender Mean (*SD*)**					**Restrained Eating Mean (*SD*)**				
	***F*_(*n* = 852)_**	***M*_(*n* = 389)_**	***t*_(1239)_**	**Sig**.	***d***	**No (*n* = 737)**	**Yes (*n* = 504)**	***t*_(1239)_**	**Sig**.	***d***
FCQ-T	124.48 (46.43)	112.78 (39.57)	4.306	<0.001	0.26	113.56 (43.77)	131.42 (43.97)	7.048	<0.001	0.41
F1	39.37(18.77)	34.75 (16.20)	4.198	<0.001	0.26	35.25 (17.62)	41.83 (18.16)	6.381	<0.001	0.37
F2	36.62 (14.20)	32.53 (13.18)	4.814	<0.001	0.29	32.81 (13.88)	39.03 (13.38)	7.869	<0.001	0.45
F3	33.05 (9.94)	31.94 (8.91)	1.891	0.059	0.11	31.94 (9.69)	33.81 (9.48)	3.362	0.001	0.19
F4	21.40 (9.29)	19.45 (8.02)	3.575	<0.001	0.22	19.57 (8.93)	22.58 (8.70)	5.888	<0.001	0.34
FCQ-T-r	47.01 (20.35)	40.65 (17.57)	5.326	<0.001	0.32	41.72 (19.27)	49.83 (19.44)	7.256	<0.001	0.42

*FCQ-T, Food Craving Questionnaire-Trait; FCQ-T-r, Food Craving Questionnaire-Trait-reduced; F1_FCQ-T_, Factor 1 of the Food Craving Questionnaire-Trait; F2_FCQ-T_, Factor 2 of the Food Craving Questionnaire-Trait; F3_FCQ-T_, Factor 3 of the Food Craving Questionnaire-Trait; F4_FCQ-T_, Factor 4 of the Food Craving Questionnaire-Trait*.

### FCQ-T-reduced

**Figure 2:**
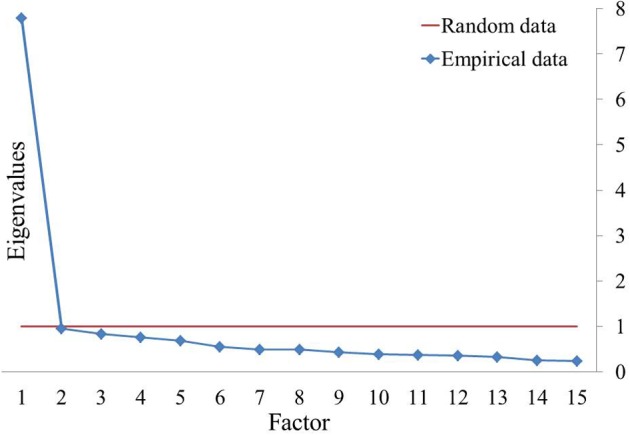
**Scree plot and parallel analysis of eigenvalues in FCQ-T-reduced**.

Just like the FCQ-T, the Kaiser-Meyer-Olkin Measure of Sampling Adequacy (KMO = 0.95) and Bartlett's Test of Sphericity [χ^2^_(105)_ = 10356.65, *p* < 0.001] indicated that the FCQ-T-reduced data were also adequate for conducting a PCA. Scree plot and parallel analysis clearly indicated a one-factor structure (Figure 2), which explained 51.93% of variance. Factor loadings and item statistics are presented in Table 5. Internal consistency was Cronbach's α = 0.93.

**Table 5:** **Factor loadings and item statistics of the food craving questionnaire trait-reduced**.

**Item**	**Factor loading**	**Description**	**Original item no**.	**Initial factor**	**Mean (*SD*)**	**r_item-total correlation_**
1	0.722	Cuando tengo deseos intensos de comer, una vez que empiezo no puedo parar. [When I crave something, I know I won't be able to stop eating once I start]	2	Lack of control	3.01 (1.84)	0.722^**^
2	0.679	A veces, cuando como lo que se me antoja, pierdo control y como demasiado. [If I eat what I am craving, I often lose control and eat too much]	3	Lack of control	3.86 (1.85)	0.687^**^
3	0.734	Sin duda alguna, las ganas de comer me hacen pensar cómo voy a conseguirlo [Food cravings invariably make me think of ways to get what I want to eat.]	5	Intentions to eat	2.98 (1.87)	0.735^**^
4	0.734	No hago más que pensar en la comida. [I feel like I have food on my mind all the time]	6	Food-related thoughts	2.29 (1.56)	0.722^**^
5	0.650	A veces me encuentro pensativo preocupado con comida. [I find myself preoccupied with food]	8	Food-related thoughts	2.58 (1.73)	0.648^**^
6	0.679	Cada vez que deseo comer algo en particular me pongo a hacer planes para comer. [Whenever I have cravings, I find myself making plans to eat]	18	Intentions to eat	3.22 (1.89)	0.685^**^
7	0.623	Siento deseos de comer cuando estoy aburrida, enfadada, o triste. [I crave foods when I feel bored, angry, or sad]	20	Negative affect	3.19 (1.85)	0.634^**^
8	0.719	No tengo la fuerza de voluntad de resistir mis deseos de comer lo que se me antoja. [I have no will power to resist my food crave]	25	Lack of control	3.54 (1.99)	0.724^**^
9	0.795	Una vez que me pongo a comer tengo problemas en dejar de comer. [Once I start eating, I have trouble stopping]	26	Lack of control	2.79 (1.87)	0.788^**^
10	0.783	Por mucho que lo intento, no puedo parar de pensar en comer. [I can't stop thinking about eating no matter how hard I try]	27	Food-related thoughts	2.30 (1.66)	0.771^**^
11	0.791	Si me dejo llevar por la tentación de comer pierdo todo mi control. [If I give in to a food craving, all control is lost]	29	Lack of control	2.53 (1.85)	0.784^**^
12	0.784	Cada vez que se me antoja una comida sigo pensando en ella hasta que me la como. [Whenever I have a food craving, I keep on thinking about eating until I actually eat the food]	31	Food-related thoughts	3.08 (1.88)	0.782^**^
13	0.779	Cuando tengo muchas ganas de comer algo estoy obsesionado con comerlo. [If I am craving something, thoughts of eating it consume me]	32	Food-related thoughts	3.02 (1.86)	0.777^**^
14	0.633	A menudo deseo comer cuando siento emociones fuertes. [My emotions often make me want to eat]	33	Negative affect	2.72 (1.79)	0.638^**^
15	0.673	Es difícil resistir la tentación de tomar comidas apetecibles que están a mi alcance. [It is hard for me to resist the temptation to eat appetizing foods that are within reach]	35	Cue-depending eating	3.91 (1.94)	0.683^**^

** *p < 0.001*.

As can be seen in Table 3, the FCQ-T-reduced was highly correlated with the FCQ-T total score and all its dimensions. In addition, the FCQ-T-reduced showed positive correlations with age (small effect size), weight cycling (from small to medium effect size), BMI (medium effect size) and food thoughts suppression (large effect size). Table 5 shows that women had higher FCQ-T-reduced scores than men and restrained eaters had higher scores than unrestrained eaters. Effect size could be considered small for gender and from small to medium for restrained eating. Finally, the FCQ-T-reduced showed a high correlation with the 24 excluded items (*r* = 0.905; *p* < 0.001).

## Discussion

The aim of the current study was to explore the factor structure, validity and reliability of the Spanish version of the FCQ-T and FCQ-T-reduced among Cuban adults. We included individuals from the general population; the majority of them were overweight and obese.

The factor structure of the Spanish version of the FCQ-T was considerably reduced in this study, which is consistent with previous results obtained with the German and Dutch versions (Nijs et al., 2007; Meule et al., 2012). However, our four-factor structure is not merely a combination of the nine original subscales.

Factor 1 included items related to the *conscious elaboration of food cravings*, which included food-related thoughts (e.g., “I spend a lot of time thinking about whatever it is I will eat next”), some guilty feelings (e.g., “I often feel guilty for craving certain foods”), intentions to eat (e.g., “Whenever I have cravings, I find myself making plans to eat”), a sense of lack of control (e.g., “If I give in to a food craving, all control is lost”), and positive reinforcement (e.g., “Sometimes, eating makes things seem just perfect”). It is important to highlight that this factor could be enough to assess food cravings, because of its large eigenvalue.

Accordingly, Factor 1 is consistent with the EI-theory, which describes the conscious experience of craving as a cycle of mental elaboration of an initial intrusive thought (May et al., 2012). This food-related thought is initially pleasurable, based on previous experiences of *positive reinforcement*, motivating the individual to elaborate it by retrieving cognitive associations and creating mental imagery of the target, which included some consummatory fantasies. Then, this “exquisite torture” (Kavanagh et al., 2005) tends to increase the intentions to obtain the desired target. Finally, guilty feelings might accompany this process. It is necessary to point out that that items of guilty feelings and food-related thoughts were also grouped together under one subscale in the German FCQ-T (Meule et al., 2012).

Factor 2 could be named as *lack of control under environmental cues*. The Spanish translation of item 4 (Table 2) could be additionally interpreted as a feeling caused by lack of control (“Detesto no poder resistir la tentación de comer”/“I hate it when I give into cravings”). Participants tended to understand this sentence as “I hate when I cannot resist the temptation of eating” and they usually focused on the following idea: “I cannot resist the temptation of eating.” Many participants emphasized this idea to surveyors while they completed the FCQ-T by saying: “Yes, I cannot resist the temptation of eating!”

Other items of this factor refer to cue-elicited eating. It has been observed that reward-related cues can instigate voluntary action to obtain such reward, through their impact on motivation and behavioral intention (Lovibond and Colagiuri, 2013). Paradoxically, it has been observed that weak temptations have a higher inhibiting effect on self-regulation processes than strong temptations (Kroese et al., 2010). Following the EI-theory, encounters with external cues, can trigger intrusive images or thoughts, starting an elaborative cycle sustained on individual motivation oriented toward the target. (May et al., 2012).

Factor 3 is linked with hedonic hunger rather than physiological hunger. Hedonic hunger substantially contributes to overeating in everyday life. As it has been suggested, delicious foods activate the neurochemical signals more potently than less tasty substances (Davis, 2013). In addition, it has been observed that food words activate eating simulations, particularly when these words refer to tempting food objects (Papies, 2013). In line with the EI-theory, the majority of items of this factor could be referred to the cognitive or physiological cues that trigger intrusive thoughts about food (May et al., 2012).

Finally, Factor 4 could be named as *eating to regulate emotions* or emotional eating, which is consistent with the five way model proposed by Macht (2008). Regarding item 37 (Table 2), although it belongs to the *positive reinforcement* subscale, its translation could also be interpreted as a feeling of relief. Additionally, emotional associations to food are the last topic that triggers food-related thoughts proposed by the EI-theory (May et al., 2012). Items from the original sub-scale of negative affect (Cepeda-Benito et al., 2000a) denote this type of triggers.

In line with other findings (Nijs et al., 2007; Vander-Wal et al., 2007; Meule et al., 2012; Crowley et al., 2014) the current results show that fewer factors than the original nine subscales of the FCQ-T can be found using PCA. It should be noted, however, that these reduced factor structures differed between studies and were different compared to the factor structure found in the current study. The four-factor structure obtained in the current study may represent how food cravings are commonly experienced in the majority of Cuban adults. In line with the EI-theory this factor structure could be divided into two broad dimensions. The first dimension is linked to the conscious elaboration of food cravings (Factor 1) and the second to *triggers* of intrusive thoughts about food (Factors 2–4).

Our results tend to confirm that the factor structure of the FCQ-T seems to be rather unstable across different versions and samples using PCA, which is supported by lower fit indices obtained from CFA in the current sample. The FCQ-T-reduced, however, showed a clear one-factor structure and high internal consistency, replicating the results obtained in the original German sample (Meule et al., 2014). Furthermore, the FCQ-T-reduced was highly correlated with the FCQ-T total score and its subscales, as well as with the excluded items. Finally, it is important to note that PCA explained a relatively small amount of variance for both the FCQ-T and the FCQ-T-reduced which suggests that the number of retained factors could be scarce.

Both the FCQ-T and FCQ-T-reduced showed very similar validity indices, as found in previous studies. That is, total scores were positively correlated with weight cycling and BMI. Although small or moderate relationships with BMI have also been found in other studies (Meule et al., 2012, 2014), BMI is not only influenced by eating behavior or food cravings, but many other factors such as genetics and environment.

With respect to weight cycling, dieters tend to experience stronger cravings that are more difficult to resist, and for the foods they are not allowing themselves to eat (Massey and Hill, 2012). For example, overweight and obese individuals with a lifetime number of weight-loss treatments more than five showed higher scores on FCQ-T than those with fewer weight-loss treatments (Fabbricatore et al., 2012).

Women and restrained eaters obtained higher scores than men and unrestrained eaters. It has been observed that women report higher levels of craving than men (Cepeda-Benito et al., 2003). In addition, obese and overweight female patients attending a low calorie diet therapy experienced more cravings for food than their male pairs (Imperatori et al., 2013). Finally, food cravings fully mediated the inverse relationship between rigid control strategies and dieting success (Meule et al., 2011) and it has been observed that chronic food restriction can trigger the desire to eat, even in the absence of hunger (Pelchat and Schaefer, 2000).

The fact that the highest correlation was found between food cravings and food-thoughts suppression brings an additional support to the EI-theory (May et al., 2012). This may be the most used strategy to control unwanted thoughts about food, but it is counterproductive (Erskine, 2008; Erskine and Georgiou, 2010). Thus, it should be assessed in future studies as an important predictor of food cravings (Barnes and Tantleff-Dunn, 2010).

The positive correlation between age and food cravings was an unexpected result. The relationship of food cravings with age might not even be strictly linear, but more complex and many factors such as gender or the effort required to achieve thought suppression could be involved. Regarding *gender*, a previous study has found that concerns about eating in men were most pronounced in the age range of 55–64 years while in women highest scores were found in those younger than 24 years (Hilbert et al., 2012). With respect to the effort required to achieve thought suppression, a recent experiment found that *suppression effort* increased linearly according to the participants' age, but *perceived difficulty* did not (Magee et al., 2014).

Another explanation for this result can be found in the extreme difficulties experienced in Cuba during the *special period* (Jiménez-Acosta et al., 2012). Taking into account the requirement of daily energy intake during 1993, the indices consumed according to age ranges were: 100% from 0 to 6 years; 90% from 7 to 13 years; 70% from 14 to 65 years and 90% in older adults (Jiménez-Acosta et al., 1998). Furthermore, the amount of energy intake exceeded the requirements for children and older adults during 1997 but barely reached 80% for youngsters and middle-aged adults (Jiménez-Acosta et al., 1998). Regarding eating, maybe the *special period* was longer and harder for youngsters and middle-aged adults. This harsh environment could condition strong food cravings in a complete generation of Cuban adults (currently older than 30 years old). It is thought that cravings for sugar and fat evolved to enhance human energy intake in unpredictable nutritional environments (Davis, 2013).

Nevertheless, the above explanation regarding the effect of the *special period* which might have conditioned an increase in food cravings in adults older than 30 years old is rather speculative and future studies should address this issue. It would be interesting to compare a Cuban cohort affected by the *special period* with the same cohort in other countries that did not experience the same difficulties regarding variables such as BMI, eating behavior and food cravings.

To summarize, both the FCQ-T and FCQ-T-reduced showed good validity indices and high internal consistency. However, the FCQ-T factor structure was significantly reduced in Cuban adults and contextual differences might have contributed to obtain this four-factor structure. Finally, the FCQ-T-reduced may represent a good alternative to efficiently assess food craving on a trait level because it showed high correlations with the FCQ-T total score as well as the rest of the items, and because its validity indices are similar to the full version.

### Conflict of interest statement

The authors declare that the research was conducted in the absence of any commercial or financial relationships that could be construed as a potential conflict of interest.

## Footnotes

^1^Some expressions like “My anxiety makes me eat” (Mi ansiedad me hace comer) could be more accurate to describe *emotional eating*. Other expressions like “I eat because of my anxiety” (Como por ansiedad) are more ambiguous and the researchers usually need to ask for its meaning.

^2^“Anxiety” could additionally mean *yearning* [see “Anxiety” (Ansiedad), a very popular song written by the Venezuelan composer José Enrique Sarabria in 1958].

^3^In Cuba each individual is regularly seen by the family physician who classifies his or her illness status.
